# The Cameroon Health Research and Evidence Database (CAMHRED): tools and methods for local evidence mapping

**DOI:** 10.1186/s12961-023-01007-4

**Published:** 2023-06-19

**Authors:** Clémence Ongolo-Zogo, Hussein El-Khechen, Frederick Morfaw, Pascal Djiadjeu, Babalwa Zani, Andrea Darzi, Paul Wankah Nji, Agatha Nyambi, Andrea Youta, Faiyaz Zaman, Cheikh Tchouambou Youmbi, Ines Ndzana Siani, Lawrence Mbuagbaw

**Affiliations:** 1 Department of Health Research Methods, Evidence and Impact, McMaster University, Hamilton, Canada; 2 Temerty Faculty of Medicine, University of Toronto, Toronto, Canada; 3 Cochrane Cameroon, Centre for Development of Best Practices in Health (CDBPH), Yaoundé Central Hospital, Yaoundé, Cameroon; 4 Nyasha Consulting, Cape Town, South Africa; 5 Department of Community Health, University of Sherbrooke, Sherbrooke, Canada; 6 The Ontario HIV Treatment Network, Toronto, Canada; 7 Department of International Health, Georgetown University, Washington, United States of America; 8 Department of Kinesiology, McMaster University, Hamilton, Canada; 9 Biostatistics Unit, Father Sean O’Sullivan Research Centre, St Joseph’s Healthcare, Hamilton, Canada; 10 Department of Global Health, Stellenbosch University, Stellenbosch, South Africa

**Keywords:** Evidence mapping, Knowledge translation, Contextualisation, Gap map analysis, Cameroon

## Abstract

**Background:**

Local evidence is important for contextualized knowledge translation. It can be used to adapt global recommendations, to identify future research priorities and inform local policy decisions. However, there are challenges in identifying local evidence in a systematic, comprehensive, and timely manner. There is limited guidance on how to map local evidence and provide it to users in an accessible and user-friendly way. In this study, we address these issues by describing the methods for the development of a centralized database of health research evidence for Cameroon and its applications for research prioritization and decision making.

**Methods:**

We searched 10 electronic health databases and hand-searched the archives of non-indexed African and Cameroonian journals. We screened titles, abstracts, and full texts of peer reviewed journal articles published between 1999 and 2019 in English or French that assess health related outcomes in Cameroonian populations. We extracted relevant study characteristics based on a pre-established guide. We developed a coding scheme or taxonomy of content areas so that local evidence is mapped to corresponding domains and subdomains. Pairs of reviewers coded articles independently and resolved discrepancies by consensus. Moreover, we developed guidance on how to search the database, use search results to create evidence maps and conduct knowledge gap analyses.

**Results:**

The Cameroon Health Research and Evidence Database (CAMHRED) is a bilingual centralized online portal of local evidence on health in Cameroon from 1999 onwards. It currently includes 4384 studies categorized into content domains and study characteristics (design, setting, year and language of publication). The database is searchable by keywords or through a guided search. Results including abstracts, relevant study characteristics and bibliographic information are available for users to download. Upon request, guidance on how to optimize search results for applications like evidence maps and knowledge gap analyses is also available.

**Conclusions:**

CAMHRED (https://camhred.org/) is a systematic, comprehensive, and centralized resource for local evidence about health in Cameroon. It is freely available to stakeholders and provides an additional resource to support their work at various levels in the research process.

**Supplementary Information:**

The online version contains supplementary material available at 10.1186/s12961-023-01007-4.

## Background

Research is an integral aspect of health systems and when used appropriately, it may inform optimal decision-making in health care and policy [1–3]. In the World Health Organisation African region (AFRO), recognition of the importance of health research for health system strengthening led to the Algiers Declaration and the Bamako Call to Action on research for health [4, 5]. Subsequently, initiatives were established to set common objectives within the region and evaluate national efforts to achieve them. These included the regional committee’s research for health ten-year strategies and the national health research systems (NHRS) barometer [3, 5].

Since its inception in 2016, the NHRS barometer has provided a comparable measure of African countries’ performance in general and within specific functional domains [3].One of these functional domains, highlights the responsibility of the NHRS for producing research and ensuring its use [3, 6, 7]. However, research productivity varies across countries, both in terms of the type of research produced and the questions addressed [8–10]. For instance, crude measures of research productivity indicate that sub-Saharan African countries continue to lag behind compared to other regions [8–11]. This may be due to challenges related to insufficient financial and technical capacity for research which is reflected in the lower number of publications from researchers affiliated with countries such as Cape Verde or Mauritania among others [8, 9]. Collaborations between international and local researchers have proven useful in tackling such challenges and helping to address research gaps in this context [10, 12, 13]. However, barriers to accessing and using the results of these research collaborations remain [13–15].

Research produced at a national level or targeting a specific setting feed into a type of evidence known as “local evidence” in the field of evidence-informed policymaking. The SUPPORT toolkit defines local evidence as

evidence that is available from the specific setting(s) in which a decision or action on an option will be taken. [ ] 'local' in this instance can refer to district, regional or national levels, depending on the nature of the policy issue being considered. Such evidence might include information on the presence of factors that modify the impacts of a policy [such as] the characteristics of an area and those who live or work in it; the need for services (prevalence, baseline risk or status); views and experiences; costs; political traditions; institutional capacity; and the availability of resources such as staff, equipment, and drugs [22].

Each of these factors lends itself to a different study design and methodology. For example, while observational studies can help to appraise and characterize the burden of a specific condition in a given population, they may not be the best type of evidence to support decisions about the choice of interventions required in a specific setting [16]. In the latter situation, other types of studies like randomized trials or cost effectiveness studies are more appropriate. However, national capacity to fund and produce such studies is not optimal in all countries and regions.

In countries with developing NHRS like Cameroon, global evidence and recommendations often fill the gaps for local evidence needed to support decision-making [17, 18]. However, contextualization or adaptation of evidence from these sources is necessary prior to implementation [17–19]. For instance, the World Health Organization produces health system guidance to support national policy development and clinical guidelines for clinical care in Cameroon [20, 21]. Prior to the release of new recommendations, contextualisation and adaptation is often conducted through multi-stakeholder development workshops. Ideally, local evidence should be at the crux of these adaptation processes as it is needed to clarify problems and burden of disease; assess intervention options; examine implementation considerations and monitor the subsequent effects [18, 22]. This local evidence can be in the form of program evaluations, costing studies, qualitative studies on views, values and preferences, community surveys, practitioners’ surveys, administrative health databases and routine program surveillance data [17, 22, 23].

A systematic approach to identifying and using local evidence is also important for contextualization [22]. However, there are challenges to finding and synthesising such evidence, as demonstrated by pilot attempts at country-specific research synthesis for Cameroon [24, 25]. Research output from Cameroon is often published in journals which are not indexed on common databases like Web of Science, Scopus or MEDLINE [25]. Furthermore, the archives of such journals are not easily searchable or accessible in some cases. Finally, there is a paucity of research being conducted on some research topics or using certain research designs; such that local evidence is simply not available [24, 25]. All these issues can be threats to systematic and comprehensive identification and use of local evidence for contextualization.

To address the challenges listed above, we propose a local evidence mapping initiative. Evidence mapping has previously been used to scope broad topic areas using evidence from a variety of sources (i.e., impact evaluations, systematic reviews, and primary research) [26–29]. More recently, this synthesis method has been used to provide clinicians and decision makers with centralized access to rapidly changing clinical guidelines and recommendations for COVID19 [30]. This methodology provides visual or graphical representations of what research is available on a specific topic, theme, policy domain or broad research question. Extensions or applications of evidence mapping complete the picture by telling us what the research says or doesn’t say. These include other synthesis methods like reviews and evidence gap maps respectively. The advantages of applying these methodologies to the Cameroonian health research system context are multifold. First, they can help ensure that research funding is not wasted by duplicating research or producing research which is not used (never read, never cited, or considered in decision making). Secondly, they can inform the development of future research priorities based on evidence gaps. Finally, they can promote timely access to local evidence needed to clarify problems or implement policies or interventions [26].

## Objectives

The objective of this study is to describe the methods for identifying and mapping local evidence through the development of a database for health research and evidence for Cameroon.

## Methods

We used evidence mapping design which combined systematic, scoping and bibliometric analysis methods to identify and categorize health literature from Cameroon to create an evidence map. Our methodology was guided by the mapping protocol established by the Global Evidence Mapping Initiative [31]. Evidence mapping involves the systematic searching and reviewing of a broad body of literature to identify knowledge and research gaps [28, 29, 31]. The results of evidence mapping can be presented in the form of visual representations (tables, graphs) and user-friendly outputs such as searchable databases [28, 29, 31].

### Searching and selecting relevant studies

#### Eligibility

##### a.Types of studies

We included quantitative (experimental, observational), qualitative, and mixed methods studies. We also included primary and secondary research focusing on health states, health outcomes, health systems, health policy, medicine, nursing and allied health professions, social determinants of health, health economics, human genetics.

##### b.Types of participants

We included studies focused primarily on Cameroonian populations.

#### Search strategy

From October 2018 to May 2019, we searched 10 electronic health databases (Excerpta Medica Database EMBASE, MEDLINE via OVID, Cumulated Index to Nursing and Allied Health Literature, CINAHL, Allied and Complementary Medicine Database AMED, Latin American and Caribbean Health Sciences Literature (LILACS), PSYCINFO, Base de Données de Santé Publique (BDSP), Archive ouverte en Sciences de l'Homme et de la Société (HAL-SHS), Base de données Persée and Erudit). We hand searched the archives of non-indexed African (African Journals Online) and Cameroonian journals (Health Sciences and Disease, Revue de Medecine et Pharmacie, Clinics in Mother and Child Health, African Journal of Integrated Health). Our search terms included Cameroon, Cameroun, Kamerun. We restricted searches to English and French articles published from 1999 to 2019. An example of our search strategy applied to EMBASE is available as a Additional file 1.

#### Selection of studies

We screened all search results and excluded ineligible studies based on title and abstracts using the Rayyan application [32]. We retrieved full-text articles of all remaining studies and screened these articles for inclusion using DistillerSR (Evidence Partners, Ottawa, Canada) [33].

### Categorizing relevant studies according to characteristics and content

#### Data extraction and management

We used DistillerSR to extract data on study characteristics presented in Table 1.

**Table 1:** Data extraction details

Study characteristic	Details
Language	English, French
Publication status	Full text publication, manuscript abstract, conference abstract or published abstract
Unique identifier	First author last name, year
Country of affiliation of the first author	Based on the location of their host institution. For first authors with multiple affiliations, the first affiliation was selected
Contact information	Email preferably
Level of access	Reported as open access or restricted
International collaboration	Defined as any co-author with a non-Cameroonian affiliation
Study location	One of the ten regions of Cameroon
Study period	Reported as the month/year at the start and end of the study
Study design	Reported as experimental (randomized controlled trials, non-randomized study of interventions), observational (case study, case series, case control, cross sectional, cohort, retrospective review), qualitative, mixed methods, and secondary analysis
Funding	Reported as public, private, self-funded, none

#### Coding scheme or taxonomy

We developed a coding scheme to label included articles and create a searchable database. The coding scheme comprised domains and subdomains guided by existing taxonomies such as the health topics used by the WHO (www.who.int/health-topics) and the Health Systems Evidence Database (healthsystemsevidence.org) at McMaster Health Forum (See Additional file 1).

Pairs of reviewers coded each article independently and resolved discrepancies by consensus. Each article could fit into multiple categories and the exercise of coding was designed to ensure that articles were allocated all relevant codes.

## Results

### Searching and selecting relevant studies

**Fig. 1:**
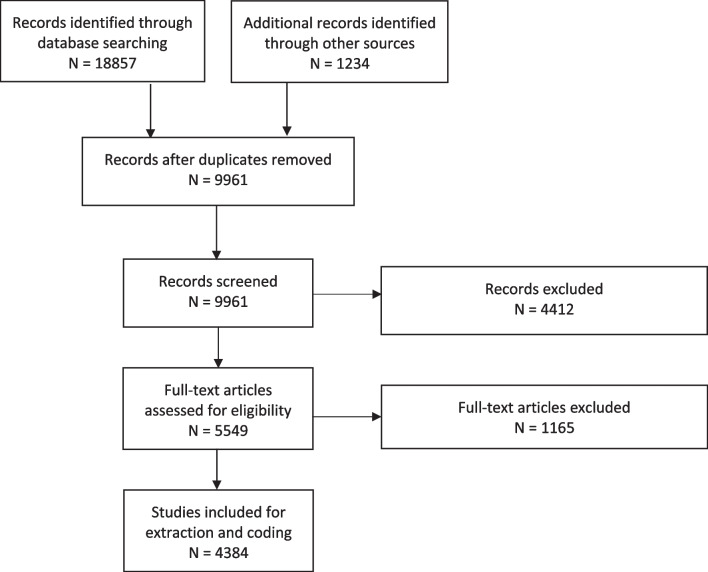
PRISMA diagram for systematic search and selection of CAMHRED studies

Our search resulted in 20,091 records. After duplicate removal, 9961 records were screened by title and abstract. We excluded 4412 and assessed 5549 full text articles for eligibility based on the inclusion criteria described above. We extracted data on 4384 eligible studies and mapped their content onto our pre-established coding scheme (Fig. 1).

### Categorizing relevant studies according to characteristics and content

#### Study characteristics

**Fig. 2:**
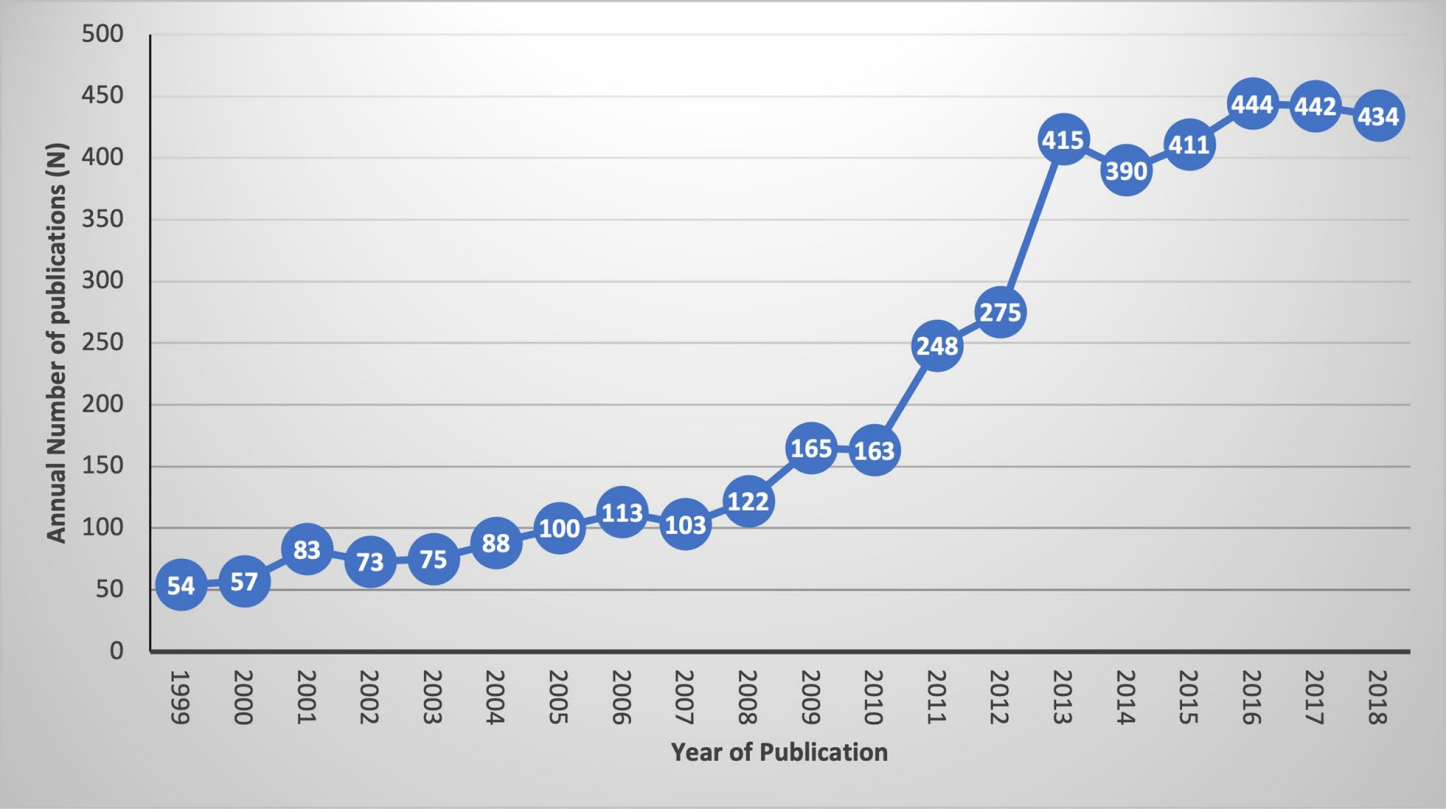
Trends in annual peer reviewed publications from 1999 to 2018

The following study characteristics were retained and made available on the database for every article: year of publication, language, study location and study design. There has been an increase in the mean annual number of peer-reviewed publications in Cameroon during our study period. Most studies in the database were published in English (*n* = 3494, 79.7%), conducted in the Centre region (*n* = 1972, 45.0%); with an observational study design (*n* = 3144, 71.7%) (See Table 2 and Fig. 2).

**Table 2:** Study characteristics

Characteristics	*N* (%)	
*Language, N (%)*		
English	3494	(79.7)
French	890	(20.3)
*Study location*		
Adamawa	195	(4.4)
Centre	1972	(45.0)
East	211	(4.8)
Far North	226	(5.2)
Littoral	747	(17.0)
North	216	(4.9)
North West	480	(10.9)
South	239	(5.5)
South West	561	(12.8)
West	331	(7.5)
Nationwide	90	(2.1)
Not reported	812	(18.6)
*Study designs*		
Experimental	250	(5.7)
Observational	3144	(71.7)
Secondary analysis	283	(6.5)
Qualitative	181	(4.1)
Mixed methods studies	88	(2.0)
Other	437	(10.0)

#### CAMHRED coding scheme or taxonomy

The full CAMHRED coding scheme or taxonomy consists of 10 main domains divided into subdomains. Each domain represents a content category used to describe the focus of research output from Cameroon. These domains include Disability, Diseases and Health Conditions, Health Systems, Medical Specialties, Pharmaceutics, Public Health, Providers, Population, Social Determinants of Health, Sexual and Reproductive Health.

The top four most coded domains in the CAMHRED were Diseases and conditions (*n* = 3524, 80.4%); Medical Specialties (*n* = 3903, 89.0%); Population (*n* = 2267, 51.7%) and Public Health (*n* = 2253, 51.4%). Within these domains, the most common subdomains were Infectious and parasitic diseases (*n* = 1194, 27.2%); Infectiology (*n* = 2005, 45.7%); Children (*n* = 654, 14.9%); Disease surveillance (*n* = 1661, 37.9%) respectively. Subdomains within the same domain were not mutually exclusive.

#### The Online Database

**Fig. 3:**
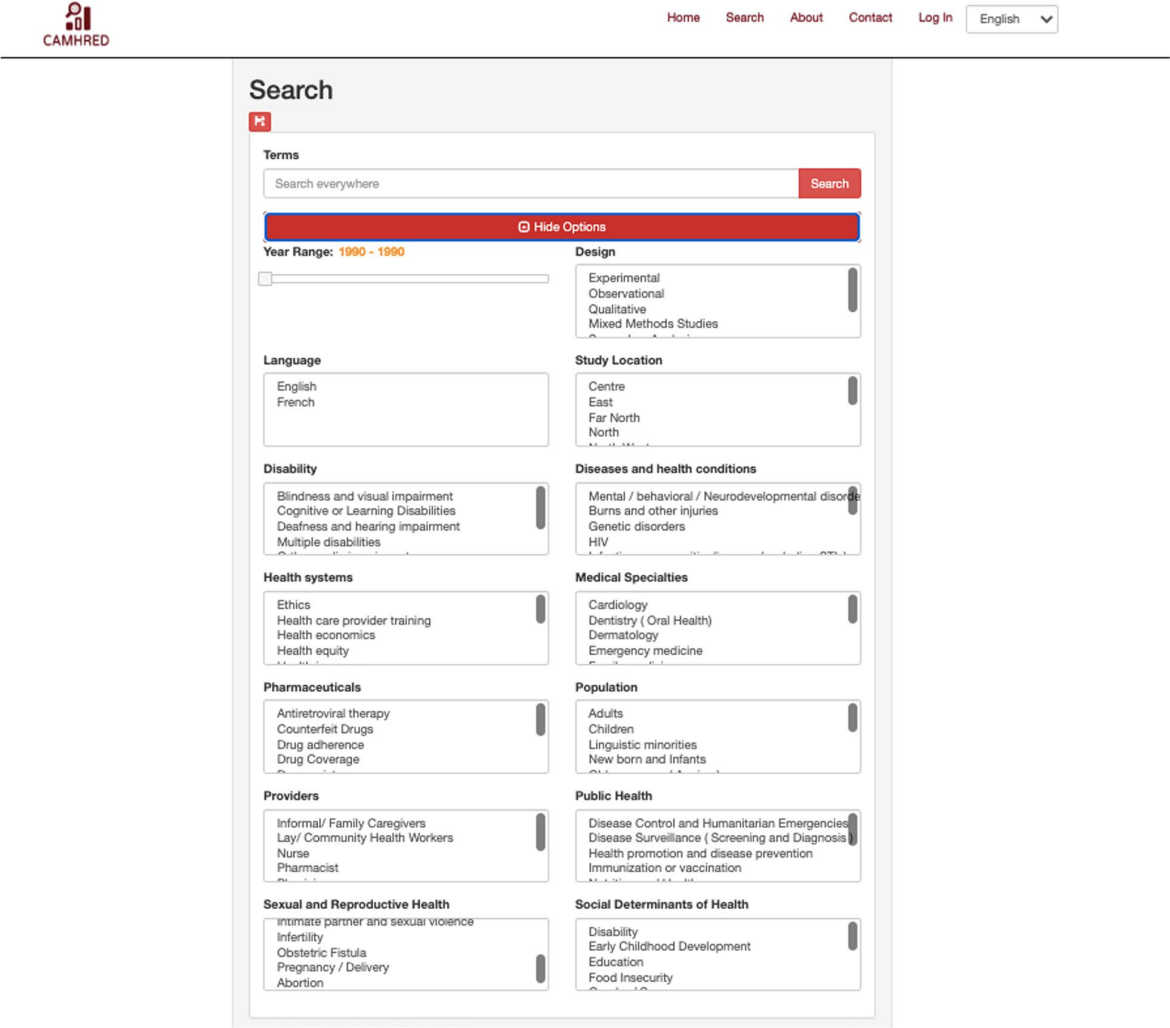
Database Guided Search Interface

Our local evidence mapping initiative produced a database of 4384 peer reviewed research articles spanning twenty years of health research in Cameroon. The first iteration of CAMHRED (https://camhred.org/) went live in December 2020 and provides a centralized and searchable online portal with studies categorized into content domains and study characteristics (The database is searchable by keywords in English or French or through a guided search. The simple search function identifies any key words in the title or abstract, including author and journal names. The advanced search function allows users to filter the results of a search based on the following features: design, setting, year, language of publication and specific content domain. Search results including abstracts, relevant study characteristics and bibliographic information are available for users to download. The layout of the guided search page is shown in Fig. 3.

## Discussion

Insights from the researchers involved in developing and using this tool as well as preliminary feedback from target users have informed this discussion about lessons learnt, strengths, limitations, future updates, and next steps for local evidence mapping in Cameroon.

### Strengths

CAMHRED is a comprehensive tool because of an extensive search strategy with sixteen databases, repositories and non-indexed journals included. Throughout the searching phase, it became evident that databases and repositories like Erudit, Persée, BDSP and HAL-SHS are useful portals to quickly identify research conducted in French or French abstracts for research published in other languages. This was important for our database as we intended on CAMHRED being a bilingual database. We noted an overlap across these French databases and other databases included in our search strategy (EMBASE, MEDLINE). This may have contributed to the large number of duplicates identified at the screening phase. We also included research published in peer reviewed journals which are currently not indexed in common electronic databases. Our experience confirmed findings from previous attempts at country-specific evidence synthesis for Cameroon which highlighted barriers to accessing local evidence housed in journals with non-searchable archives [25]. We hand searched four such journals with relevant health research in Cameroon and included them into the database. This will help users identify literature they would have had to previously hand search. Therefore, CAMHRED can also play a role in conducting timely yet comprehensive literature searches and reviews targeting health in Cameroon. Since the launch of the database, one of such journals (Health Sciences and Disease) has enabled an electronic search of its archives which will facilitate our next update.

This study is a steppingstone for other local evidence mapping initiatives in Cameroon and countries with similar national health research systems. The applications of such initiatives are dependent on target user categories (funders, decisionmakers, students, researchers) and their objectives (research or funding prioritisation, knowledge translation, local planning or decision making, literature reviews for primary or secondary research). For instance, knowledge brokering organizations active at the interface between research and decision making in Cameroon, have described using evidence mapping to provide context-specific policy options and inform priority setting exercises [34, 37–39]. Partnerships between the CAMHRED team and such organisations could help them meet the time-sensitive demands of their work while retaining or even improving on a systematic approach to identifying and using local evidence. In addition, the experience from Cameroon can also help establish similar initiatives in other countries by leveraging existing networks and relationships such as the Cochrane African Network (especially its Francophone hub with headquarters in Cameroon) [40]. Ultimately, tools like CAMHRED and capacity building in local evidence mapping can also contribute to improving the production and use of research for health in countries which have already shown commitment to improving their national health research systems.

Our work also contributes to the growing literature on the methodology to guide evidence mapping which is currently marred by lack of consensus and the absence of guidelines such as those present for systematic reviews [28, 29]. To the best of our knowledge, there are no mapping protocols for local evidence, specifically. While we followed and modified the GEM initiative protocol [31] to fit our objectives, we also had to blend systematic, scoping and bibliometric analyses techniques to identify and characterise broad research areas from a specific setting (Cameroon). Thus, the CAMHRED protocol provides preliminary guidance on adapting existing global evidence mapping methods to support local evidence mapping and gap analysis.

### Challenges

Developing CAMHRED was not without challenges. As the first iteration of a country-specific database spanning 20 years; the breadth of health-related topics and research output to review, categorize and describe was large. Our approach and methods were both resource-intensive and time-intensive posing challenges for the first iterations of country-specific databases elsewhere. It took us a year from our last searches to coding completion and another 6 months before the database was ready for our first online launch. Other resource implications included software license purchases, staff time and IT costs. For instance, our initial search strategy resulted in thousands of articles which needed to be deduplicated prior to screening. Software such as Rayan and DistillerSR were instrumental in such de-duplication while research staff and volunteers contributed several hours to screening, data abstraction and coding. While the resulting product is comprehensive in content; feasibility and sustainability should be considered seriously prior to engaging in similar initiatives in other countries. In terms of sustainability, frequent updates to the database’s content are expected with a timeline model, based on our experiences with the first iteration. We anticipate that the process of updating the database should take anywhere between 3 and 6 months, provided there is commitment from at least two pairs of reviewers. Preparations for our next iteration of the database are currently underway with an updated search and new features. We will be revising our search strategy to reduce duplication from overlapping databases as seen in our first iteration. We are also exploring automation tools to reduce the burden of manual screening and classification [41].

Most content categories in CAMHRED were based on existing taxonomies (WHO health topics, McMaster Health Forum Health Systems Evidence Database) and categories from one domain, (sexual and reproductive health) were inspired by stakeholder priorities. In other words, input from target users outside the research team was not included during the development of this taxonomy. This decision was based on time constraints and the assumption that these existing classification schemes were widely known and accepted. However, understanding of the domains and subdomains were expected to influence user experience and usefulness of the database. This was confirmed by preliminary user feedback following our first launch. To address these concerns, a thorough and detailed version of our user guide will be included in the next update as well as opportunities for training workshops with target users. We will also explore consensus and stakeholder engagement options for the addition of new topics to the database.

### Next steps

The next update of CAMHRED’s content and user interface is scheduled for 2022. In this new version, we hope to address preliminary user feedback such as: keyword search sensitivity; user interface (colors, accessibility); access to full text sources; and the availability of RIS file downloads. New features to expect include an expanded taxonomy (new COVID-19 domain) and a resources page (tutorials, webinars, user guides templates for mapping products and completed maps). We are also working on establishing formal partnerships with research and policy platforms such as Cochrane Cameroon with the following objectives: increasing stakeholder buy-in; setting up a request and rapid response mechanism for evidence maps and gap maps; and formal evaluations.

Our next steps include publishing guidance on how to use CAMHRED to create extensions of evidence mapping such as gap maps. We will be using a case study on local evidence mapping and gap analysis for sexual and reproductive health to describe how CAMHRED can inform decisions regarding future research and policy.

## Conclusion

Harnessing the increasing research output from Cameroon to help inform decisions made locally by clinicians, policymakers and patients is even more relevant now given rapidly changing global recommendations in a pandemic context. CAMHRED provides a one-stop shop for understanding what local evidence already available and existing gaps. However, it is not intended as a substitute for other evidence sources and comprehensive search strategies for systematic reviews, evidence briefs, rapid reviews, and responses. Instead, we suggest using the database as a tool to ease and support discussions surrounding problem definitions and implementation considerations for evidence-informed decision-making within a Cameroonian context. The methods we describe here can be tailored to settings with similar NHRS and beyond.

### Supplementary Information

**Additional file 1.** An example of our search strategy applied to EMBASE.

## Figures and Tables

**Fig. 4:**
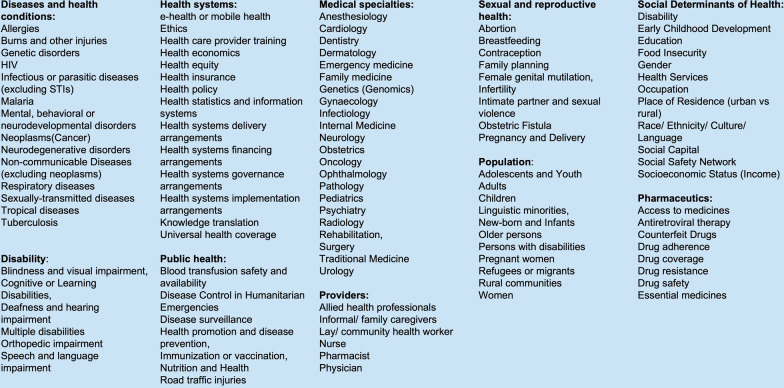
CAMHRED Taxonomy domains and subdomains

## Data Availability

Not applicable.

## Appendix 1: Taxonomy

See Fig. 4.
