# Supplementary material for: The Cameroon Health Research and Evidence Database (CAMHRED): tools and methods for local evidence mapping
Source: Health Res Policy Syst. 2023 Jun 19;21:58. doi: 10.1186/s12961-023-01007-4 (PMC10278273; doi:10.1186/s12961-023-01007-4)
Supplement: Supplementary file 1 — Additional file 1. An example of our search strategy applied to EMBASE. [file 12961_2023_1007_MOESM1_ESM.docx]

| **Date of search** | 18.05.2019 |
| --- | --- |
| **Database** | Embase 1996 to 2019 and Ovid MEDLINE(R) 1996 to2019 |
| **# of Hits** | 8471 |
| **Searched by** | COZ |
| **Provider** | OVID |
| **Publication date / Date range** | ______1999_______ − ______2019________ |
| **Search terms** | Kamerun OR Cameroon OR Cameroun |
| **Search string** | (Kamerun or Cameroon or Cameroun).mp. [mp=title, book title, abstract, original title, name of substance word, subject heading word, floating sub-heading word, keyword heading word, organism supplementary concept word, protocol supplementary concept word, rare disease supplementary concept word, unique identifier, synonyms] |
| **Language** | English French German |
| **Other limits** | Humans |
| **How search terms were searched (Keyword? Full text? Ect.)** | keyword |
| **Other comments** |  |
